# Increasing Visual Search Accuracy by Being Watched

**DOI:** 10.1371/journal.pone.0053500

**Published:** 2013-01-02

**Authors:** Yuki Miyazaki

**Affiliations:** 1 School of Psychology, Chukyo University, Nagoya-shi, Aichi, Japan; 2 Research Institute of Visual Sciences, Kanagawa University, Yokohama-shi, Kanagawa, Japan; University of Rome, Italy

## Abstract

In daily life, huge costs can arise from just one incorrect performance on a visual search task (e.g., a fatal accident due to a driver overlooking a pedestrian). One potential way to prevent such drastic accidents would be for people to modify their decision criterion (e.g., placing a greater priority on accuracy rather than speed) during a visual search. The aim of the present study was to manipulate the criterion by creating an awareness of being watched by another person. During a visual search task, study participants were watched (or not watched) via video cameras and monitors. The results showed that, when they believed they were being watched by another person, they searched more slowly and accurately, as measured by reaction times and hit/miss rates. These findings also were obtained when participants were videotaped and they believed their recorded behavior would be watched by another person in the future. The study primarily demonstrated the role of being watched by another on the modulation of the decision criterion for responding during visual searches.

## Introduction

In daily life, huge costs can arise from just one failing performance during what is essentially a visual search task (e.g., a fatal accident due to a driver overlooking a pedestrian, or an airline hijacking resulting from overlooking dangerous materials during baggage screening). A potential way to prevent such drastic costs would be to shift the decision criterion during the visual search. In the car driving situation, for example, the potential for an accident could be decreased by making a slower, more careful search in favor of speed or quickness. The principal aim of the present study was to manipulate the decision criterion for responding during visual searches by creating an awareness of being watched by another person.

People are highly sensitive to the signals of being watched [1]–[6], which affect their attention [7]–[10] and cognition ([11]–[13], for reviews, [14], [15]). Further, it has been reported that an awareness (or subawareness) of being watched (or observed: e.g., [16]) affects the perceiver's criteria for a making decisions regarding cooperative or prosocial behavior [17]–[28].

To date, no study has investigated the role of the being watched by another person on the modulation of a decision criterion in a non-social context. This study examines whether an awareness of being watched affects participants' performances during visual searches. There are two plausible hypotheses as to the criterion shift during visual searches with reference to a speed-accuracy tradeoff [29], [30]. First is that participants will search quickly but inaccurately (that is, priority is placed on speed). In contrast, second is that they will search slowly and accurately (that is, priority is placed on accuracy).

In summary, the present study examines the effect of being watched by another person during visual searches. If the first hypothesis is supported, reaction times (RT) will be faster and errors more frequent under the condition of being watched (watched condition) than the condition of being unwatched (unwatched condition). If the second hypothesis is supported, RTs will be longer and errors fewer under the watched than the unwatched condition.

## Participants

Seventy-three paid graduates and undergraduates participated in the present experiments. There were 24 participants in Experiment 1 (10 males and 14 females; mean age  = 20.88 years, *SD*  = 2.59), 24 different participants in Experiment 2 (8 males and 16 females; mean age  = 20.21 years, *SD*  = 1.59), and 25 different participants in Experiment 3 (9 males and 16 females; mean age  = 20.28 years, *SD*  = 1.93). All participants were unaware of the purpose of the experiment, and had normal or corrected to normal eyesight.

Ethical approval for this study was obtained from the Research Ethics Committee of the School of Psychology in Chukyo University. All participants provided written informed consent. They gave permission to use their data in the analysis.

## Experiment 1

### Methods

#### Apparatus

The visual stimuli were projected on a 17 inch CRT monitor (BenQ G775 with a resolution of 1024×768 and refresh rate of 60 Hz). To collect the RTs, we used a response time box [31]. Stimulus presentation and data acquisition were controlled by a Windows XP-based computer (Hewlett-Packard xw4600) running Matlab 2007b with Psychtoolbox [32], [33]. To suppress the ambient sounds in the laboratory, participants were provided white noise through headphones (SONY MDR-XB700). To arrange the watched condition, we used two digital video (DV) cameras (SONY HDR-PJ40V) with tripod stands connected to two 10 inch LCD monitors (Logitec LCM-T102AS). The cameras were connected to the LCDs via standard RCA-type cables and up-scan converters (Princeton PUC-AVBOXL).

#### Design

There were two within-participant factors: being watched (watched condition, unwatched condition) and target presence on the visual search task (target present trials, target absent trials), which were crossed to yield four conditions.

**Figure 1:**
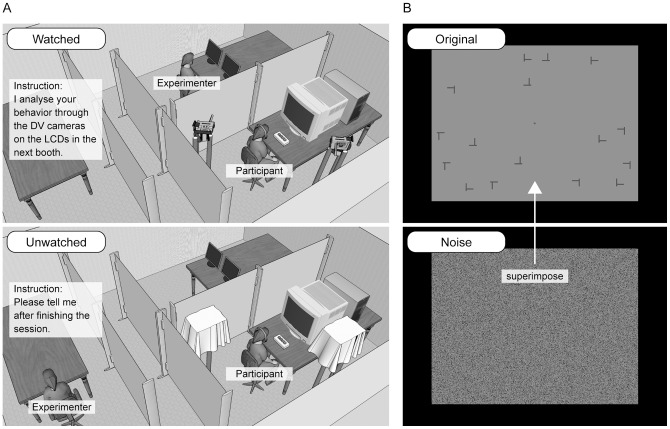
Illustrations of the experimental settings and stimuli. (A) The top picture represents the watched condition and the bottom picture represents the unwatched condition. (B) An example of the target present trials. Participants were required to search for a T in any orientation from among offset Ls in any orientation. The salt-and-pepper visual noise (bottom picture) was superimposed on the search display (top picture).

In the watched condition (top on Figure 1A), participants were watched by the experimenter (who was the author) through the DV cameras on the LCDs. One DV camera was positioned on the right front side of the participants and the other was to the left side behind them (the distance from the participants to each of the DV cameras was 65 cm). The participants were instructed that the experimenter would watch and analyze their behavior during the sessions in the next booth (however, they were watched but their behavior was not being analyzed). They were not able to see the experimenter because each booth was separated by partitions.

In the unwatched condition (bottom on Figure 1A), the two DV cameras were covered by opaque fabrics. The participants were provided the instruction that they would need to tell the experimenter after finishing the session.

#### Stimuli

The search display consisted of 16 stimuli, a central fixed indicator (a cross), and salt-and-pepper visual noise (Figure 1B). The stimuli were dark-gray (approximately 13.73 cd/m^2^) rotated Ts (0°, 90°, 180°, and 270°) and similarly rotated offset Ls (0°, 90°, 180°, and 270°) with a visual angle of 0.95×0.95 degrees, presented on a mid-gray (approximately 23.95 cd/m^2^) background with a visual angle of 25.39×19.04 degrees. The stroke width was 0.13 degrees. The Ts and the offset Ls were targets and distracters, respectively. The stimuli were presented on an imaginary 8×6 grid except for the four cells around the center of the grid. Each cell size was 3.17×3.17 degrees. Each stimulus was centered within the cell and randomly jittered from 0 to 0.79 degrees. In the target present trials, a single target and fifteen distracters were presented. In the target absent trials, only sixteen distracters were presented. A dark-gray fixed cross (0.32×0.32 degrees) was used to indicate the center of the display. The black (approximately 9.02 cd/m^2^) salt-and-pepper noise (proportion of noise density was 0.4) was superimposed on the display to reduce the visibility of the stimuli and to induce inaccurate performance.

#### Procedure

The experiment was composed of four sessions (two watched and two unwatched conditions). To counterbalance the order of the being watched or not, 12 participants had sessions in the following order: watched–unwatched–watched–unwatched, and then the remaining 12 participants experienced the reverse order. The first and second sessions were practice sessions and were not included in the statistical analyses. Each session began with 18 practice trials with correct/incorrect feedback after each response prior to 72 test trials, half of which were target present trials. In order to induce inaccurate performances, the participants were not provided feedback during the 72 test trials, and the preceding 18 practice trials were excluded from statistical analysis.

Each session started when participants pressed a response button on an instruction screen. After a fixed cross was presented on the center of the display for 1,000 ms, the search display appeared, and remained until a response was made or for 10,000 ms. The participants were instructed to make a target present or absent judgment by using two response buttons, as quickly and accurately as possible. Twelve participants were required to press the left button for a target present judgment, and the right button for target absent; the remaining twelve participants had the reverse setup (left button for target absent and right button for target present). After the response selection or 10,000 ms, the next trial began. In the preceding 18 practice trials, visual correct/incorrect feedback was provided at the center of the display after the response selection or 10,000 ms. The participants were instructed to maintain the viewing distance (57 cm), although they were free to move their heads. The experiment was conducted in a well-lighted room (horizontal illuminance on the desk was approximately 620 lx).

### Results

Trials in which the participants did not make a response for 10,000 ms were removed from the statistical analyses (0.52% of trials were removed). Errors during visual searches were divided into groups: “miss” (target absent judgment when target present) and “false-alarm” (target present judgment when target absent).

#### Reaction Times

**Figure 2:**
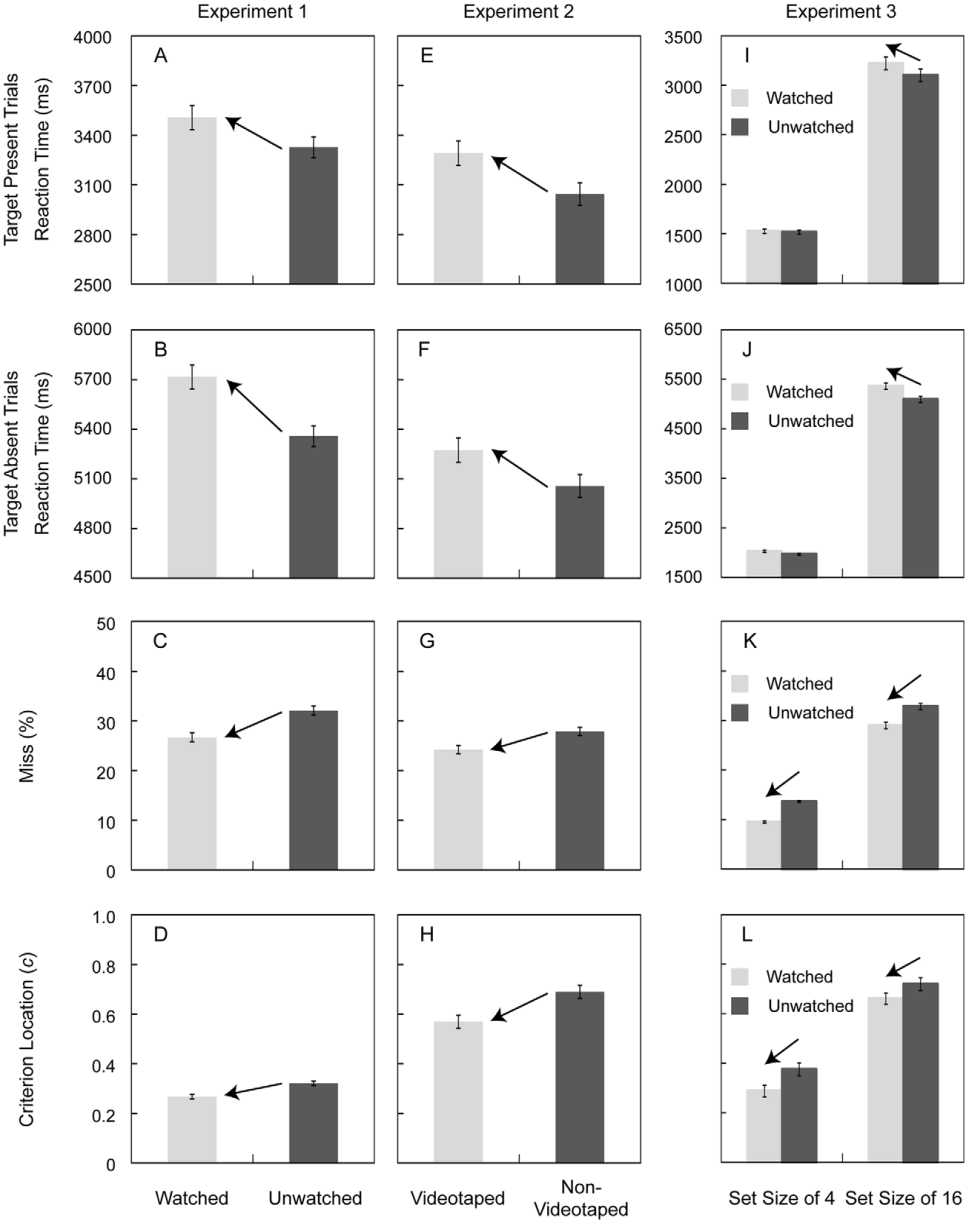
Results in this study. (A)–(D) Results in Experiment 1. (E)–(H) Results in Experiment 2. (I)–(L) Results in Experiment 3. Error bars represent the within-participant standard error of mean [37].

Trials on which the participants made an error were discarded from RT analysis. Means of RTs under the target present and absent trials are shown in Figures 2A and 2B. A two-way repeated measures analysis of variance (ANOVA) revealed a significant main effect of being watched (*F*(1, 23)  = 15.94, *p*<.001, *η*_p_^2^ = .41) and that of target presence (*F*(1, 23)  = 270.61, *p*<.001, *η*_p_^2^ = .92). Since there was a significant difference in the interaction between the factors (*F*(1, 23)  = 4.38, *p*<.05, *η*_p_^2^ = .16), tests of simple main effect were conducted to confirm the influence of being watched on the RT. The tests revealed a significant difference between the watched and unwatched conditions in the target present trials (*F*(1, 23)  = 5.55, *p*<.03, *η*_p_^2^ = .19), and in the target absent trials (*F*(1, 23)  = 18.58, *p*<.001, *η*_p_^2^ = .45). These results indicate that the reaction times under the watched condition were slower than the unwatched condition. To confirm that outliers did not cause the differences in RT (which was defined as RT > mean +2*SD,* or as RT < mean – 2*SD*), the statistical analyses were repeated using the trimmed means of RTs. Since the overall patterns of statistical results were similar to the results using non-trimmed RT means, we rejected the possibility that outliers influenced the differences in RTs.

#### Accuracy

Since the means of false-alarm rates (number of false-alarm trials divided by the total number of the target present trials) were extremely low and did not vary much between conditions (2.18% of watched and 1.68% of unwatched), we focused only on the miss rates.

The means of miss rates (number of miss trials divided by the total number of the target absent trials) are shown in Figure 2C. A paired *t*-test was applied to the miss rates after an arc-sine transformation, and the result revealed a significant difference in the main effect of the being watched (*t*(23)  = −2.91, *p*<.01, *r* = .52). The results showed that the miss rates under the watched condition were lower than the unwatched condition.

#### Sensitivity and Criterion Location

For each participant and for each condition, *d'* (sensitivity) and *c* (criterion location) parameters were calculated from hit (target present judgment when target was present) and false-alarm rates on the basis of signal detection theory. Where necessary, the proportions of 1 and 0 (i.e., perfect accuracy) were adjusted to avoid infinite values ([34], p.8). For the means of *c* (Figure 2D), a paired *t*-test revealed a significant main effect of being watched (*t*(23)  = −2.54, *p*<.02, *r* = .47), showing that *c* was negatively shifted under the watched condition compared to the unwatched condition (a negative direction represents a tendency to respond “target present”). For the means of *d'*, the *t*-test did not reveal a significant difference between the conditions (*t*(23)  = 1.69, *p*>.10, *r* = .33). The mean ± *SD* of *d'* in the watched condition was 2.68±0.61 and in the unwatched condition 2.52±0.37.

### Discussion

The results indicated that the participants searched more slowly and accurately when they thought they were being watched by another person, supporting the second hypothesis: the participants prioritized accuracy. In addition, with reference to signal detection theory, it found that the effect of being watched did not alter the sensitivity (*d'*) but only the criterion (*c*) during visual searches.

To further test the effect of being watched during visual searches, in Experiment 2 we set up the condition that the participants were being videotaped by DV cameras during visual searches. Participants were instructed that the videotaped data would be used for behavioral analyses after finishing the experiment (that is, we created an awareness of “being watched in future”). This experiment was conducted to examine whether participants would search slowly and accurately while being watched in non-real (future) time.

## Experiment 2

### Methods

#### Apparatus

The apparatus was identical to Experiment 1 except we removed the two LCD monitor displays and their associated cables and equipment.

#### Design

There were two within-participant factors: being videotaped (videotaped condition, non-videotaped condition) and target presence on the visual search task (target present trials, target absent trials), which were crossed to yield four conditions.

In the videotaped condition, participants were videotaped by the DV cameras during the session. In the non-videotaped condition, the two DV cameras were covered by opaque fabrics and were not operated. The locations of the DV cameras were the same as for Experiment 1. In both conditions, the experimenter stayed in the separate booth (same location as unwatched condition in Experiment 1, see the bottom on Figure 1A). The participants were required to tell the experimenter when they had finished their session. In the videotaped condition, they were further provided the instruction that the experimenter would analyze the participants' behaviors after finishing the experiment, on the basis of the videotaped data (however, the experimenter did not do that).

#### Stimuli

The stimuli were identical to Experiment 1.

#### Procedure

The procedure was almost identical to Experiment 1. To counterbalance the order of the being videotaped or not, 12 participants completed sessions in the following order: videotaped–non-videotaped–videotaped–non-videotaped conditions, and then the remaining 12 participants completed the sessions in the reverse order.

### Results

Trials on which the participants did not make a response for 10,000 ms were removed from the statistical analyses (0.75% of trials were removed).

#### Reaction Times

Trials in which the participants made an error were discarded from RT analysis. The means of RTs for the target present (or absent) trials are shown in Figures 2E and 2F). A two-way repeated measures ANOVA revealed a significant main effect of being videotaped (*F*(1, 23)  = 12.01, *p*<.01, *η*_p_^2^ = .34) and that of target presence (*F*(1, 23)  = 211.06, *p*<.001, *η*_p_^2^ = .92). There was no significant difference in the interaction between the factors (*F*(1, 23)  = 0.14, *p*>.71, *η*_p_^2^ = .01). These results showed that reaction times under the videotaped condition were slower than the non-videotaped condition. To confirm that outliers did not cause the differences in RT (which was defined as RT > mean + 2*SD,* or as RT < mean – 2*SD*), the statistical analyses were repeated using the trimmed means of RTs. Since the overall patterns of statistical results were similar to the results using non-trimmed RT means, we rejected the possibility that outliers influenced the differences in RTs.

#### Accuracy

The means of false-alarm rates were extremely low and did not vary systematically with each condition (2.63% for the watched condition and 3.31% for the unwatched condition).

The means of miss rates are shown in Figure 2G. A paired *t*-test was applied to the miss rates after an arc-sine transformation, and the test revealed a significant main effect of being videotaped (*t*(23)  = −2.22, *p*<.04, *r* = .42). The results showed that the miss rates in the videotaped condition were lower than the non-videotaped condition.

#### Sensitivity and Criterion Location

Where necessary, the proportions of 1 and 0 were adjusted to avoid infinite values. For the means of *c* (Figure 2H), a paired *t*-test revealed a significant main effect of being videotaped (*t*(23)  = −2.27, *p*<.04, *r* = .43), suggesting that *c* was negatively shifted under the videotaped condition compared to the non-videotaped condition. For the mean of *d'*, the *t*-test did not reveal a significant difference between both conditions (*t*(23)  = 1.23, *p*>.23, *r* = .25). The mean ± *SD* of *d'* in the videotaped condition was 2.73±0.75 and in the non-videotaped condition it was 2.61±0.60.

### Discussion

The results indicated that the participants searched more slowly and accurately when they were being videotaped and they believed their behavior would be watched in future. Results also indicated that the non-real-time signals of being watched also affected the decision criterion during visual searches. Taken together, the results of Experiments 1 and 2 showed that the signals of being watched by another person, whether in real time or in the future, contribute significantly to a decision criterion shift of prioritizing accuracy over speed during visual searches.

However, these conclusions have one caveat: The slower RTs and fewer misses under the watched (or videotaped) condition might be a part of the post-search process rather than the search process (i.e., the participants were careful in making their response selection, after the visual search). The next experiment was conducted to examine this possibility. In Experiment 3, we manipulated the number of stimuli on the search display (i.e., the set size) and measured RTs as a function of set size. Changes in the slope of the RT × set size search function would reflect an increment in RT caused by adding a stimulus to the search display (see, e.g., [35]). Whereas, the changes in the “*y*-intercept” of the search function reflect the increment in RT under pre-/post-search process (e.g., [36]).

If the finding that participants searched more slowly and accurately when they were being watched was derived from the change in the search process, then the slope of the search function would be steeper under the watched condition compared to the unwatched condition.

## Experiment 3

### Methods

#### Apparatus

The apparatus was identical to Experiment 1.

#### Design

There were three within-participant factors: being watched (watched condition, unwatched conditions), set size (stimulus search set size of 4, 16), and target presence on visual search task (target present trials, target absent trials), which were crossed to yield eight conditions.

#### Stimuli

The stimuli were identical to Experiment 1. In the condition with a set size of 4, a single target and three distracters (or only four distracters) appeared on the target present (or absent) trials. The condition with a set size of 16 was the same as Experiment 1.

#### Procedure

The procedure was identical to Experiment 1 except for the number of sessions and trials. This experiment was composed of three sessions. To counterbalance the order of the being watched or not, 12 participants completed the sessions in the following order: watched–unwatched–watched, and then the remaining 13 participants completed the sessions in the order: unwatched–watched–unwatched. The first session was a practice session and was not included in the statistical analyses. Each session started with 16 practice trials with correct/incorrect feedback for each response prior to 128 test trials (half of which had target present). The 16 practice trials were excluded from the statistical analyses.

### Results

As before, trials on which the participants did not make a response for 10,000 ms were excluded from statistical analyses (0.17% of trials were removed).

#### Reaction Times

Trials in which participants made an error were discarded from RT analysis. The means of RTs in the target present (or absent) trials are shown in Figures 2I and 2J).

A three-way repeated measures ANOVA revealed a significant main effect of being watched (*F*(1, 24)  = 7.38, *p*<.02, *η*_p_^2^ = .24), that of set size (*F*(1, 24)  = 454.19, *p*<.001, *η*_p_^2^ = .95), and that of target presence (*F*(1, 24)  = 313.11, *p*<.001, *η*_p_^2^ = .93). There were significant differences in the interactions between the being watched condition × set size (*F*(1, 24)  = 4.90, *p*<.04, *η*_p_^2^ = .17), and between set size × target presence (*F*(1, 24)  = 360.27, *p*<.001, *η*_p_^2^ = .94). No other significant interaction was found. Tests of simple main effects were conducted to confirm the influence of being watched on RT in each set size. The tests revealed a significant difference between the watched and unwatched conditions with the set size of 16 (*F*(1, 24)  = 6.70, *p*<.02, *η*_p_^2^ = .22), whereas there was no significant difference between both conditions with the set size of 4 (*F*(1, 24)  = 2.05, *p*>.16, *η*_p_^2^ = .08). The lack of the significant difference in the set size of 4 may be explained by the following analysis of the search function's slope and *y*-intercept.

**Figure 3:**
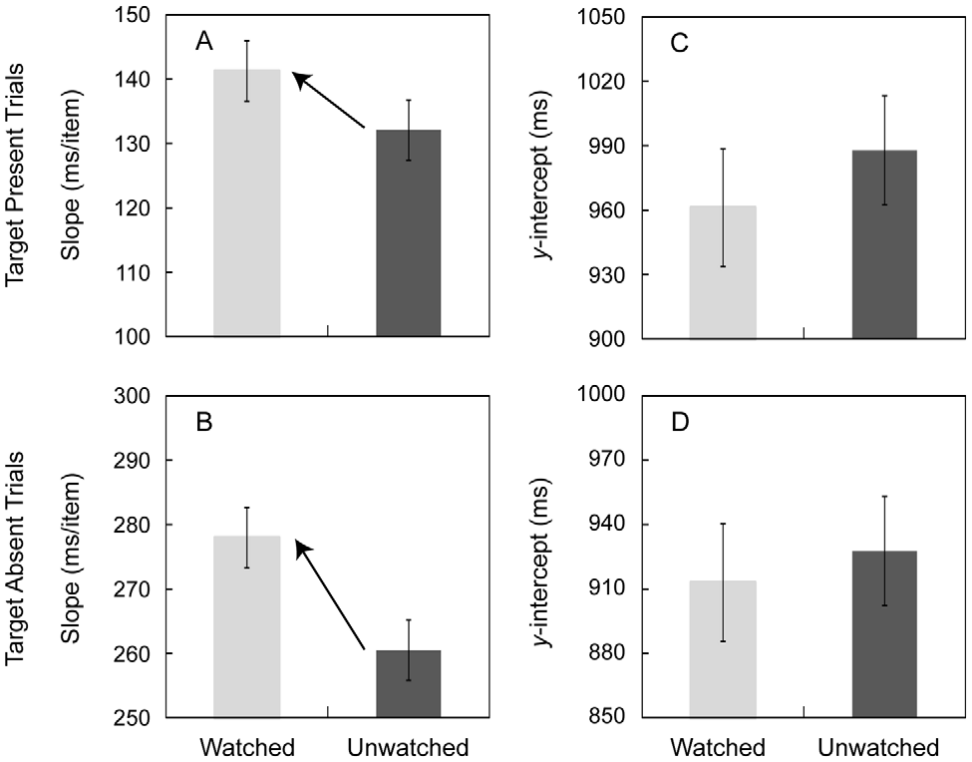
Results in Experiment 3. (A) Means of search function's slope (in ms/item) in the target present trials. (B) Means of the slope in the target present trials. (C) Means of search function's *y*-intercept (in ms) in the target present trials. (D) Means of the intercept in the target present trials. Error bars represent the within-participant standard error of mean [37].

The slope and *y*-intercept were calculated for each participant and in each condition. For the means of the slope (Figures 3A, 3B), a two-way repeated measures ANOVA revealed a significant main effect of being watched (*F*(1, 24)  = 4.90, *p*<.04, *η*_p_^2^ = .17) and of target presence (*F*(1, 24)  = 360.27, *p*<.001, *η*_p_^2^ = .94). There was no significant difference in the interaction between those factors (*F*(1, 24)  = 0.44, *p*>.51, *η*_p_^2^ = .02). These results suggested that the slope in the watched condition was steeper than in the unwatched condition. For the means of the *y*-intercept (Figures 3C, 3D), no significant main effects or interactions were obtained by a two-way repeated measures ANOVA (being watched condition: *F*(1, 24)  = 0.37, *p*>.54, *η*_p_^2^ = .02; target presence: *F*(1, 24)  = 2.46, *p*>.12, *η*_p_^2^ = .09; interaction: *F*(1, 24)  = 0.02, *p*>.88, *η*_p_^2^ = .001).

Finally, to confirm that outliers did not cause the difference in RTs and the slope, the statistical analyses were also conducted by using the trimmed means of RTs. Since the overall patterns of statistical results were similar to the results using non-trimmed RT means, we rejected the possibility that outliers influenced the differences in RTs.

#### Accuracy

The means of false-alarm rates were extremely low and did not vary systematically with each condition (2.13% and 2.38% for set sizes of 4 and 16, respectively, in the watched condition, and 1.88% and 2.57% for set sizes of 4 and 16 in the unwatched condition).

The means of miss rates are shown in Figure 2K. A two-way repeated measures ANOVA was applied to the miss rates after an arc-sine transformation, and then the ANOVA revealed a significant main effect of being watched (*F*(1, 24)  = 5.80, *p*<.03, *η*_p_^2^ = .19) and that of set size (*F*(1, 24)  = 91.80, *p*<.001, *η*_p_^2^ = .79). There was no significant difference in the interaction between the factors (*F*(1, 24)  = 0.0004, *p*>.98, *η*_p_^2^ = .00002). In agreement with Experiment 1, these results suggested that the miss rates in the watched condition were lower than the unwatched condition.

#### Sensitivity and Criterion Location

Where necessary, the proportions of 1 and 0 were adjusted to avoid infinite values. For the means of *c* (Figure 2L), a two-way repeated measures ANOVA revealed a significant main effect of being watched (*F*(1, 24)  = 5.91, *p*<.03, *η*_p_^2^ = .20) and that of set size (*F*(1, 24)  = 160.77, *p*<.001, *η*_p_^2^ = .87). There was no significant difference in the interaction between the factors (*F*(1, 24)  = 0.50, *p*>.48, *η*_p_^2^ = .02). These results suggested that *c* was negatively shifted under the watched condition. For the means of *d'*, an ANOVA revealed a significant main effect of set size (*F*(1, 24)  = 86.03, *p*<.001, *η*_p_^2^ = .78), and then no other significant interaction was found. The means ± *SD* of *d'* in the set sizes of 4/16 in the watched condition were 3.26±0.72/2.57±0.65, and in the unwatched condition 3.21±0.63/2.41±0.69.

### Discussion

The results showed that the slope of the search function was steeper for the watched condition than the unwatched condition, but the *y*-intercept remained constant between the two conditions. These results indicated that the decision criterion shift during visual searches induced by the signal of being watched occurred in the search process. In addition, the results of Experiment 1 were replicated; the trends of the parameters (RT, misses, false-alarms, *d'*, and *c*) were consistent with those in Experiment 1. The results of Experiment 3 emphasize the contention that participants search more slowly and accurately when they are being watched by another person.

## General Discussion

The overall aim of the present study was to manipulate the decision criterion during visual searches by making participants aware of being watched by another person. Experiments 1 and 3 showed that participants searched more slowly and accurately when they were being watched (compared to when they were unwatched) by another person. Although it has already been shown that the signals of being watched (or observed) affect participants' criteria for making a decision on social tasks (e.g., [23]), the present study demonstrated for the first time that those signals can also affect the decision criteria on non-social visual search tasks. These findings suggest possibility that decision criteria under all contexts (social or otherwise) could be influenced by signals about being watched by another person. It would be interesting to investigate the effect of being watched during other non-social tasks, such as object recognition tasks.

Experiment 2 showed that participants searched more slowly and accurately when they were being videotaped, and they believed their recorded behavior would be watched by another person in the future. Also, a decision criterion shift during visual searches was induced by non-real-time signals of being watched. The results of Experiment 2 have significant implications with regard to the car driving example, given above; installing in-vehicle cameras and being videotaped at all times during driving could contribute to a driver's more careful visual searching. Although this most likely would be considered impractical on a widespread basis, it may be of interest to transportation companies, i.e., employers of truck drivers, bus drivers, etc.

In comparison to the present study using video-monitoring setups for the condition of being watched, more subtle cues of being watched (e.g., eye-like paintings or dots: [23]–[28], photocopied eyes: [17], [18], [20]–[22]) have been used in previous studies. This difference leads to a possibility that participants might also search more slowly and carefully when the eye-like paintings or photocopied eyes are superimposed on the background of the search display. It is worth considering the application possibilities in daily life because the cost and inconvenience of such an approach would be much smaller than with videotape-monitoring equipment.

Finally, three potential problems in the present study need to be mentioned. Firstly, we imposed a considerably difficult search task on the participants; salt-and-pepper visual noise was superimposed on the search display and knowledge of results feedback was not provided after each response during test trials to induce inaccurate performance. Therefore, the decision criterion shift during visual searches when the participants were being watched by another might be limited to the difficultly of the search task (e.g., similar to searching for a man wearing a dark suit while driving a car in the rain). It is suggested that future studies should investigate this issue by manipulating the level of task difficulty. Next, in the visual search task in Experiment 3, we only used two set sizes (set size of 4 and 16), whereas most previous visual search studies have manipulated more than three set sizes. It is suggested that in order to assure the robustness of the finding indicating that the slope of the search function is steeper in the watched than in the the unwatched condition, additional studies of visual search using over three set sizes should be undertaken. Finally, there is the possibility that the results were influenced by the social status (or power) difference between the experimenter and the participants. All the participants in this study were undergraduate, or graduate students at the institute where the experimenter was an assistant professor. Moreover, some participants were the experimenter's students. Therefore, the experimenter's social status could have been higher than that of the participants. This difference in social status might have enhanced the effect of being watched. It is suggested that control studies with using participants and experimenters with equal status, such as by hiring undergraduate students to be experimenters for example, should be undertaken in the future.

In conclusion, the present study primarily demonstrated the role of being watched on the modulation of a decision criterion during visual searches. Of course, the author does not suggest that people need to perform searches slowly and accurately in all circumstances. However, some visual searches ideally should be performed as carefully as possible to prevent fatal accidents and huge costs associated with possible visual search failures. The results of this study could have a significant impact under the condition that people should search carefully.

## References

[1] Conty L, Tijus C, Hugueville L, Coelho E, George N (2006) Searching for asymmetries in the detection of gaze contact versus averted gaze under different head views: A behavioural study. Spat Vis 19: 529–545 doi: 10.1163/156856806779194026. 17278526 10.1163/156856806779194026

[2] Doi H, Ueda K (2007) Searching for a perceived stare in the crowd. Perception 36: 773–780 doi: 10.1068/p5614. 17624121 10.1068/p5614

[3] Palanica A, Itier RJ (2011) Searching for a perceived gaze direction using eye tracking. J Vis 11: 1–13 doi: 10.1167/11.2.19. 10.1167/11.2.19 PMC3933318 21367758

[4] Senju A, Hasegawa T (2006) Do the upright eyes have it? Psychon Bull Rev 13: 223–228. 16892985 10.3758/bf03193834

[5] Senju A, Hasegawa T, Tojo Y (2005) Does perceived direct gaze boost detection in adults and children with and without autism? The stare-in-the-crowd effect revisited. Vis Cogn 12: 1474–1496 doi: 10.1080/13506280444000797.

[6] von Grünau M, Anston C (1995) The detection of gaze direction: A stare-in-the-crowd effect. Perception 24: 1297–1313 doi: 10.1068/p241297. 8643334 10.1068/p241297

[7] Conty L, Gimmig D, Belletier C, George N (2010) The cost of being watched: Stroop interference increases under concomitant eye contact. Cognition 115: 133–139 doi: 10.1016/j.cognition.2009.12.005. 20070959 10.1016/j.cognition.2009.12.005

[8] Miyazaki Y, Ichihara S, Wake H, Wake T (2012) Attentional bias to direct gaze in a dot-probe paradigm. Percept Mot Skills 114: 1007–1022 doi: 10.2466/21.07.24.PMS.114.3.1007–1022. 22913037 10.2466/21.07.24.PMS.114.3.1007-1022

[9] Senju A, Hasegawa T (2005) Direct gaze captures visuospatial attention. Vis Cogn 12: 127–144 doi: 10.1080/13506280444000157.

[10] Yokoyama T, Ishibashi K, Hongoh Y, Kita S (2011) Attentional capture by change in direct gaze. Perception 40: 785–797 doi: 10.1068/p7003. 22128551 10.1068/p7003

[11] George N, Driver J, Dolan RJ (2001) Seen gaze-direction modulates fusiform activity and its coupling with other brain areas during face processing. NeuroImage 13: 1102–12 doi: 10.1006/nimg.2001.0769. 11352615 10.1006/nimg.2001.0769

[12] Mason MF, Hood BM, Macrae CN (2004) Look into my eyes: gaze direction and person memory. Memory 12: 637–643 doi: 10.1080/09658210344000152. 15615320 10.1080/09658210344000152

[13] Vuilleumier P, George N, Lister V, Armony J, Driver J (2005) Effects of perceived mutual gaze and gender on face processing and recognition memory. Vis Cogn 12: 85–102 doi: 10.1080/13506280444000120.

[14] George N, Conty L (2008) Facing the gaze of others. Neurophysiol Clin 38: 197–207 doi: 10.1016/j.neucli.2008.03.001. 18539254 10.1016/j.neucli.2008.03.001

[15] Senju A, Johnson MH (2009) The eye contact effect: mechanisms and development. Trends Cogn Sci 13: 127–134 doi: 10.1016/j.tics.2008.11.009. 19217822 10.1016/j.tics.2008.11.009

[16] Wedekind C, Milinski M (2000) Cooperation through image scoring in humans. Science 288: 850–852. doi: 10.1126/science.288.5467.850. 10.1126/science.288.5467.850 10797005

[17] Bateson M, Nettle D, Roberts G (2006) Cues of being watched enhance cooperation in a real-world setting. Biol Lett 2: 412–414 doi: 10.1098/rsbl.2006.0509. 17148417 10.1098/rsbl.2006.0509 PMC1686213

[18] Bourrat P, Baumard N, McKay R (2011) Surveillance cues enhance moral condemnation. Evol Psychol 9: 193–199. 22947966 10.1177/147470491100900206 PMC10480913

[19] Burnham TC, Hare B (2007) Engineering human cooperation. Does involuntary neural activation increase public goods contributions? Hum Nat 18: 88–108 doi: 10.1007/s12110-007-9012-2. 26181843 10.1007/s12110-007-9012-2

[20] Ekström M (2012) Do watching eyes affect charitable giving? Evidence from a field experiment. Exp Econ 15: 530–546 doi: 10.1007/s10683-011-9312-6.

[21] Ernest-Jones M, Nettle D, Bateson M (2011) Effects of eye images on everyday cooperative behavior: a field experiment. Evol Hum Behav 32: 172–178 doi: 10.1016/j.evolhumbehav.2010.10.006.

[22] Francey D, Bergmüller R (2012) Images of eyes enhance investments in a real-life public good. PLoS One 7: e37397 doi: 10.1371/journal.pone.0037397. 22624026 10.1371/journal.pone.0037397 PMC3356250

[23] Haley KJ, Fessler DMT (2005) Nobody's watching? Subtle cues affect generosity in an anonymous economic game. Evol Hum Behav 26: 245–256 doi: 10.1016/j.evolhumbehav.2005.01.002.

[24] Keller J, Pfattheicher S (2011) Vigilant self-regulation, cues of being watched and cooperativeness. Eur J Pers 25: 363–372 doi: 10.1002/per.797.

[25] Mifune N, Hashimoto H, Yamagishi T (2010) Altruism toward in-group members as a reputation mechanism. Evol Hum Behav 31: 109–117 doi: 10.1016/j.evolhumbehav.2009.09.004.

[26] Oda R, Niwa Y, Honma A, Hiraishi K (2011) An eye-like painting enhances the expectation of a good reputation. Evol Hum Behav 32: 166–171 doi: 10.1016/j.evolhumbehav.2010.11.002.

[27] Powell KL, Roberts G, Nettle D (2012) Eye images increase charitable donations: evidence from an opportunistic field experiment in a supermarket. Ethology 118: 1–6 doi: 10.1111/eth.12011.

[28] Rigdon M, Ishii K, Watabe M, Kitayama MS (2009) Minimal social cues in the dictator game. Journal of Economic Psychology 30: 358–367 doi: 10.1016/j.joep.2009.02.002.

[29] Schouten JF, Bekker JAM (1967) Reaction time and accuracy. Acta Psychol 27: 143–153 doi: 10.1016/0001-6918(67)90054-6. 10.1016/0001-6918(67)90054-6 6062205

[30] Wickelgren WA (1977) Speed-accuracy tradeoff and information processing dynamics. Acta Psychol 41: 67–85 doi: 10.1016/0001-6918(77)90012-9.

[31] Li X, Liang Z, Kleiner M, Lu ZL (2010) RTbox: a device for highly accurate response time measurements. Behav Res Methods 42: 212–225 doi: 10.3758/BRM.42.1.212. 20160301 10.3758/BRM.42.1.212

[32] Brainard DH (1997) The Psychophysics Toolbox. Spat Vis 10: 433–436 doi: 10.1163/156856897X00357. 9176952

[33] Pelli DG (1997) The VideoToolbox software for visual psychophysics: Transforming numbers into movies. Spat Vis 10: 437–442 doi: 10.1163/156856897X00366. 9176953

[34] Macmillan NA, Creelman CD (2005) Detection Theory: A User's Guide 2nd ed. Mahwah NJ: Lawrence Erlbaum Associates.

[35] Wolfe JM (2003) Moving towards solutions to some enduring controversies in visual search. Trends Cogn Sci 7: 70–76 doi: 10.1016/S1364-6613(02)00024-4. 12584025 10.1016/s1364-6613(02)00024-4

[36] Woodman GF, Vogel EK, Luck SJ (2001) Visual search remains efficient when visual working memory is full. Psychol Sci 12: 219–274 doi: 10.1111/1467-9280.00339. 11437304 10.1111/1467-9280.00339

[37] Cousineau D (2005) Confidence intervals in within-subject designs: A simpler solution to Loftus and Masson's method. Tutorial in Quantitative Methods for Psychology 1: 42–45.

